# Abstracts From JADPRO Live at APSHO 2015

**Published:** 2016-01-01

JW MARRIOTT DESERT RIDGE, PHOENIX, ARIZONA NOVEMBER 5–8, 2015

## JL301. Adherence to Oral Cancer Therapies in the Adult Oncology/Hematology Patient

Katherine Mitchell, APRN, AOCNP, Shana Cassady, APRN, AOCNP, and Whitney Perry, APRN, AOCNP; CBC Group, Louisville, KY

There has been an increase in the development of oral cancer agents in recent years. This has created a paradigm shift in the management of the adult oncology/hematology population as patients must assume increased responsibility for understanding and adhering to the prescribed plan. An area of interest for the Oncology Nursing Society (ONS) and the American Society of Clinical Oncology (ASCO) is oral chemotherapy adherence. The Quality Oncology Practice Initiative (QOPI) sets standards for certification of oncology practices based upon key outcome measures. One area of focus is improving continuity of care and adherence for patients prescribed oral agents. A team of advanced oncology nurses at a QOPI-certified institution created a standardized process to monitor adherence in adult oncology/hematology patients taking oral oncolytic medications. We explored: 1) To what degree do patients adhere to the prescribed schedule when taking oral agents; and 2) What percentage of patients require an intervention by the healthcare team due to side effects or patient concerns? The team developed a standardized process to monitor patients prescribed oral agents. This included ensuring timely acquisition of drug and investigating possible roadblocks to payment, providing appropriate and timely education regarding dosing and side effects, and creating a telephone call system. To assess adherence, the validated eight-item Morisky Medication Adherence Scale (MMAS) was utilized to ensure standardization and to prompt routine follow-up phone calls with patients. Patients were called two weeks after initiating therapy, and either two weeks following the first call or two weeks after follow-up in the office. Discussion included tolerance, concerns, and side effects. This communication determined the need for intervention. Analysis of information obtained from phone calls showed a "high level adherence" (MMAS score 8/8) of 82% (N=162) on the first follow-up call, and 86% (N=123) on the subsequent call. Seventeen percent of patients required an intervention to address patient concerns or side effects. The MMAS score was independent of the need for intervention, with no correlation identified based on MMAS scores. A consistent systemic approach is necessary to assess adherence in patients taking oral oncolytics. There appears to be no correlation between self-reported adherence and the need for clinician intervention. Our findings suggest that follow-up phone calls and clinician assessment may be an effective approach to identify which patients need support with adhering to therapy. Examination of MMAS scoring and the link to intervention is an area of interest for future study.

## JL302. The CARE (Clinical Assessment and Rapid Evaluation) Clinic: Improving the Oncology Patient Experience and Outcomes

Mary Jane LaRoche, MS, ANP-BC, AOCNS, ACHPN, Whitney Herter, PA-C, MHS, Sarah Eppers, RN, BSN, OECN, Nicole Giles, RN, BSN, OCN, Adam Poust, PharmD, James Bachman, Tom Purcell, MD, MBA, and Wells Messersmith, MD, FACP; University of Colorado Cancer Center, Aurora, CO

Oncology patient visits are anticipated to increase annually as the population ages with an estimated 24,540 new cancer diagnoses in Colorado in 2015. This year, University of Colorado Anschutz Cancer Pavilion (ACP) visits have increased by 14%. Chemotherapy dose intensity impacts patient outcomes, especially treatment with curative intent, with symptom management helping achieve this oncology quality indicator. Timely and appropriate symptom management is vital for cancer patients. Limited same day appointment availability in oncology clinics leads to patients being inappropriately directed to the emergency department (ED) to address issues such as fever, pain, and gastrointestinal symptoms that can be managed outpatient. In 2014, the ACP established the need for an advanced practice provider (APP)-led symptom management clinic. Baseline data collected from ED, inpatient admissions, and outpatient infusion visits along with National Comprehensive Cancer Network (NCCN) sites with similar clinics, reinforced the case for an acute symptom management clinic to address increased patient volumes. In February 2015, the CARE Clinic opened with limited hours staffing two APPs and an RN sharing busy infusion center resources. The global aim is to provide evidence-based symptom management to oncology patients with acute and chronic cancer and treatment related symptoms through improved access and quality care. Clinic goals include providing value-based care, avoiding unnecessary ED visits, reducing inpatient length of stay and readmissions, provision of multidisciplinary care, and enhancing cancer patient experiences and outcomes. By April clinic hours expanded fulltime on weekdays. Resource utilization, referral sources, basic demographics, cancer type, chief complaint, diagnoses, billing level and disposition (including ED avoidance and direct admissions) are tracked to help characterize patients utilizing services. Patient and internal customer experiences are surveyed with future plans to utilize the Edmonton Symptom Assessment System (ESAS-r). These metrics reveal opportunities to improve oncology care through the development of evidence-based guidelines with cancer specific pathways. Patient surveys and staff feedback have been very positive. Quality improvement with PDSA (Plan-Do-Study-Act) cycles to refine clinic capabilities and needs is ongoing. By six months, CARE Clinic services reached over 450 patient visits with 21% patient ED avoidance including direct admission.

## JL303. Local Observational Study of Supportive Care, Treatment and Bone Health in Prostate Cancer Patients Receiving Androgen Deprivation Therapy (LOSST trial)

Phyllis C. Everett, NP-C, Lynchburg Hematology Oncology, Lynchburg, VA

*Background:* Advanced practice nurses (APRNs) caring for prostate cancer patients in a community setting noted that bone health was not being monitored in patients receiving ADT. A review of published articles in early 2011 revealed little attention to the topic so a study was designed, then approved by the local IRB, the first initiated by an all APRN group. Purpose: To determine if attention to bone health in prostate cancer patients on ADT reduced the incidence of skeletal related events (SRE), loss of BMD and/or affected progression of disease determined by measurement of prostate specific antigen (PSA). Sample: A convenience sample of men 18 years or older in a community urology clinic with non-metastatic prostate cancer with planned ADT for at least one year. *Methods:* Patients were approached during routine visits to the urology clinic regarding participation. Baseline vitamin D and bone mineral density (BMD) study with DEXA scan were performed on all participants. Subjects were managed by a protocol for vitamin D supplementation and/or treatment of osteopenia/osteoporosis as needed. DEXA scan was repeated at two years. PSA was also monitored at least twice per year. A total of 28 men were enrolled and divided into 3 groups. Group 1 (2 men) received bisphosphonate plus vitamin D, Group 2 (17 men) received Vitamin D and calcium supplementation only, Group 3 (9 men) received standard of care. Additional data collected included age, weight/body mass index (BMI), Gleason score, smoking history, and reporting of SREs. *Results:* Group 1 – Bisphosphonate plus vitamin D-both men 2/2 (100%) showed increased BMD at two years. One/2 (50%) developed bone metastasis during the study. Group 2 – Vitamin D/calcium treatment alone-showed 3/17 (17.6%) with increased BMD, 14/17 (82%) had decreased BMD (> 3%) at 2 years. Four/17 (23.5%) developed bone metastasis, one died as a result of his disease. Group 3 showed 1/9 (11%) with increased BMD and 8/9 (89%) decreased. One/9 men developed bone metastasis, 1/9 (11%) developed a second primary. Three/28 (11%) men had increase in PSA. Target vitamin D levels were achieved in 9/28 (32%) subjects. Three/9 (33%) of those who reached the therapeutic vitamin D level developed bone metastasis. One/28 (4%) did not complete the second vitamin D screening. *Conclusions:* The addition of bisphosphonates and vitamin D to ADT treatment may improve BMD; the effect on delay/prevention of development of SREs is unknown. More attention to vitamin D supplementation could be helpful in maintaining or improving BMD in patients on ADT. *Implications/Significance:* Limited enrollment did not allow for a statistically significant result, however, this is useful data as a pilot study. The study was helpful in the development of a protocol for assessment of vitamin D and BMD in the urology clinic and improved care of patients.

## JL304. Evaluation of the Oncology Nurse Practitioners Web Education Resource Course

Margaret Quinn Rosenzweig, PhD, FNP-BC, AOCNP, University of Pittsburgh School of Nursing, Pittsburgh, Pennsylvania, Sara Klein, MS, BSN, RN, Marcus Evans, San Diego, California, Mary Connolly BSN, RN, DMC Sinai-Grace, Detroit, Michigan, and Rose Hoffmann, PhD, RN, University of Pittsburgh School of Nursing, Pittsburgh, PA; funding source: National Cancer Institute (1 R25 CA148050-01A1)

*Background:* The Oncology Nurse Practitioner Web Education Resource (ONc-PoWER) is an online course developed by Margaret Rosenzweig, PhD, FNP-BC, AOCNP, from the University of Pittsburgh School of Nursing and funded by the National Cancer Institute (1 R25 CA148050-01A1). The course is designed specifically for Nurse Practitioners (NPs) in their first year of oncology practice paired with an on-site mentor (physician, nurse practitioner or physician assistant). Based on the Oncology Nursing Society’s Competencies for Entry to Practice, the course consists of 5 interactive modules: 1) the new patient visit 2) presenting a patient with cancer 3) cancer visits across the continuum of care 4) palliative and hospice care and 5) self-care and professional development. The purpose of this study was to examine to what degree the learning objectives were met; the NPs and Mentors comments about content learned and areas for course improvement were evaluated. *Method:* NPs and mentors completed the course over 4–6 months then completed a course evaluation. There are 6 items on the course evaluation with Likert scaled responses of 1) did not meet objective 2) somewhat met objective 3) met objective 4) more than met objective 5) exceeded objective expectation. *Results:* Enrollment is ongoing. Thirty NPs new to practice and 22 oncology mentors have completed evaluations thus far. Responses overall are favorable.

**Table 1:**
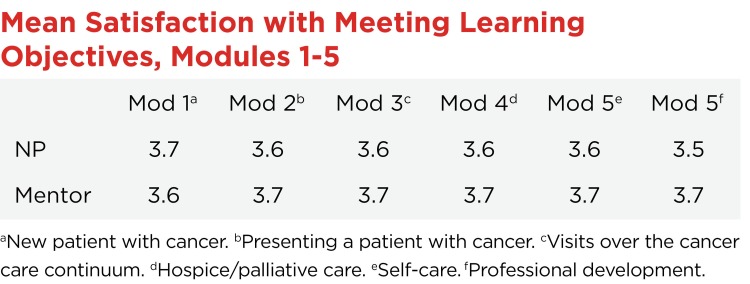
Mean Satisfaction with Meeting Learning Objectives, Modules 1-5

*Conclusion:* The ONc-PoWER web enhanced oncology orientation program was favorably evaluated by nurse practitioners new to cancer care and their mentors. Areas most often identified by the novice NPs as new content learned were using National Comprehensive Cancer Network (NCCN) guidelines for practice and "how to organize a new patient visit." Areas for improvement were identified as the need for more complex information overall, more specific information regarding malignancies and more in depth information regarding (chemotherapy/treatment) side effects and oncologic emergencies. This method of electronic orientation could standardize the exposure of essential basic cancer care competencies at entry to oncology NP practice.

## JL305. Bringing Quality Into Your Practice

Laura Mulka, APRN, AOCN, ACHPN, Connecticut Multispecialty Group, Wethersfield, CT

As professionals, we pride ourselves on delivering high quality care. However, until five years ago, there was no method of demonstrating a practice’s level of excellence distinction from competing practices. In 2010, the Quality Oncology Practice Initiative (QOPI) was launched. QOPI recognizes practices that deliver the highest quality of care by reviewing twenty standards including over one hundred quality measures. In addition, there is an onsite review to observe nursing procedures and examine policies and processes. Over the past five years, not only have the standards changed, but the bar has been raised to attain these goals. The Quality Oncology Practice Initiative is a preview of impending changes. The Oncology Care Model (OCM) was developed by the Center for Medicare and Medicaid Innovation. This is a new payment and delivery system, which is based on quality of care. In the near future, practices that demonstrate a commitment to improve care and lower costs for their patients will receive financial incentives. It is predicted that the private pay insurance companies will follow this lead. Demonstrating excellence in care is not only essential for our patients, but will soon become crucial in the financial survival of practices. The QOPI site review involves twenty standards, with multiple subsections. The major areas include: treatment planning, staff training and education, chemotherapy orders and drug preparation, patient consent and education, safe chemotherapy administration, and monitoring and assessment of patient well being. This poster will highlight the oral chemotherapy standard and the assessment of the patient’s psychosocial standard. These are considered the most challenging criteria to meet. The oral chemotherapy standard requires an extremely detailed process. The measure entails education regarding the agent, including side effects and schedule of the medication. It also necessitates the creation of a formal process in the office. This is to assure that the patient obtains the prescription, is compliant taking the medication, has regular follow up visits, and has interventions for side effects. Documentation has to be extremely specific in this category. Assessing the patient’s psychosocial well being is also a very challenging standard. The requirements include an evaluation with each chemotherapy treatment, either by the provider or nurse, to assess the physical, social, spiritual and psychological well being of the patient. If the patient has concerns, interventions must be documented. This includes referrals to allied health professionals, supportive services or other interventions. The Advanced Practice Provider must be aware of not only the changes and implementation in the demand for high standards, but also the financial implications of their documentation. Advanced Practice Providers take on the principle role in not only providing direct patient care, but also in creating policies and processes to ensure that the highest quality of care is accessible for their patients. The paradigm of quality monitoring and improvement is now shifting to the outpatient setting. We need to be prepared to undertake this challenge and lead the way for excellence in the office.

## JL306. Oncology Nurse Communication Training Needs Across the Cancer Trajectory

Jo Hanson, RN, MSN, CNS, OCN, City of Hope National Medical Center, Duarte, CA, Elaine Wittenberg, PhD, University of Kentucky, Lexington, KY, Betty Ferrell, PhD, MA, RN, FAAN, FPCN, CHPN, City of Hope National Medical Center, Duarte, CA, and Denice Economou, RN, MN, CNS, CHPN, City of Hope National Medical Center, Duarte, CA; funding source: National Cancer Institute

*Scope of Problem:* Understanding care needs across the cancer trajectory requires sensitive communication about diagnosis, discussing factors influencing treatment decision-making, mediating family communication, and psychosocial counseling about difficult topics. Oncology nurses, especially Advanced Practice nurses (APRN), are expected to initiate shared decision-making with the patient and family; however there is little instruction available for nurses on how to communicate in a way that accomplishes this approach.*Purpose:* To assist oncology nurses with discussions about care topics, we received funding from the National Cancer Institute to provide a two-day communication training course offered annually for four years to a cohort of 100 competitively selected oncology nurses. *Methods:* Participants in our first course (n = 82) completed a pre-course survey to report educational offerings at their institution, the perceived effectiveness of communication with patients at their institution across the cancer trajectory, and their own degree of difficulty with communication topics. *Results:* More than 75% of teams reported that their organization had not provided training on how to break bad news, talk about goals of care and care transitions, or talk about recurrence. Nurses rated communication with patients about survivorship (4.36), end of life (4.64), during bereavement (3.92) and time of death (4.72) as least effective at their institution (on a scale of 0 = not effective to 10 = very effective); and their own communication about hospice and palliative care (5.18), conflicts with patient/family (5.18), conflict with team members (4.87), and financial topics (4.08) as most difficult (on a scale of 0 = not difficult to 10 = very difficult). More than 50% of nurses surveyed are present when bad news or prognosis information is provided to the patient. *Implications for Practice:* This nationwide training provides nurses with education to improve communication with patients and families across the cancer trajectory. *Conclusion:* This communication training curriculum focuses on understanding the patient narrative, including the family caregiver, and providing psychosocial and spiritual care. Care needs across the cancer trajectory communication training for oncology nurses is needed, especially given their strong impact on quality patient care and family support.

## JL307. The Advocacy Connector: Bridging the Gap

Joanne Vanak, RN, MSN, Janssen Biotech, Washington, DC, Ellen Ivey, BS, Washington, DC, and John Kerrane, MS, RPh, New York, NY

*Objective:* There is a knowledge gap between the capabilities of advocacy groups and healthcare providers (HCPs) and patients. The need to create awareness about valuable resources, available through not-for-profit organizations, for HCPs, patients, and caregivers led to the creation of the "Advocacy Connector" (www.advocacyconnector.com). This web-based resource links patients and HCPs to a variety of advocacy groups that serve to address a host of patient needs (e.g. emotional, educational and financial support). This resource will focus on the area of immunology, oncology, and a rare disorder, Castleman’s disease. This resource has been recognized as a valuable resource for HCP and their practices. Simply providing the Advocacy Connector link (www.advocacyconnector.com) to patients had generated valuable discussion regarding some of the needs, gaps, and challenges that patients experience as they embark on their cancer journey. *Methods:* Not-for-profit state and national level advocacy groups focusing on services directed towards patients across the United States were given a survey to complete. Information regarding state-level advocacy groups was gleaned from all 50 states. Initially, individuals within the groups were asked if they would participate in an on-line resource that would highlight the capabilities of their respective organizations. The advocacy groups identified their key services, the results of which were then reviewed and validated by an independent vendor. *Results:* The Advocacy Connector is a compilation of information from both state and national level advocacy groups, specifically focused on immunology and the following cancer-related diseases: prostate, ovarian, breast, colorectal, lung, leukemia, lymphomas, melanoma, myeloma, pancreatic, sarcoma, Waldenström’s macroglobuemia, and a rare disorder, Castleman’s disease. Advocacy Connector is organized so that patients and HCPs can easily access information about each advocacy group according to the type of services they provide for each type of cancer. Available information includes: help-lines, alternative and complementary therapies, research, caregiver support, clinical trial information, counseling, end-of-life care, financial assistance, legal/insurance assistance, men’s health, pain management and palliative care, screening and early detection, spiritual support, survivorship, travel services, veteran services, wellness, nutrition and exercise, women’s health, and young adult cancer support. Materials may be printed for home-use by patients and caregivers. Telephone numbers for the resources are included in the printed information for those who have no access to computers. Information is also available in non-English languages (e.g. Spanish). *Conclusion:* We believe the Advocacy Connector will help bridge the knowledge gap by increasing awareness of the extent of services offered by oncology advocacy groups and thereby increase patient knowledge, access to services, and overall satisfaction. Metrics have determined an increased need to have capabilities and resources identified for patients and caregivers; the addition of individual state resources has been acknowledged by patients through surveys to be an invaluable additional resource as they search for advocacy organizations within their local areas.

## JL308. Utilization of Cancer Screening Guidelines by APRNs

Joyce Dains, DrPH, JD, RN, FNP-BC, FNAP, FAANP, Department of Nursing, The University of Texas MD Anderson Cancer Center, Houston, TX

*Introduction/Purpose:* Utilization of established cancer screening guidelines ensures recommendations that are individualized to a patient’s level of risk. The purpose of this survey was to understand how APRNs in Texas access and utilize current cancer screening guidelines. *Methods:* The instrument was investigator developed with 21 multiple choice response items. To ensure validity, the items were based on review of the literature and previous work. Internal consistency of the responses had a Cronbach’s alpha coefficient 0.89, indicating high reliability. *Results:* From 2525 randomly selected APRNs in Texas, 367 completed the survey, for a response rate of 14.5%. Over 70% of the respondents worked in private practice (42.7%), community or public health clinic (17.7%), or hospital-based outpatient clinic (13.2%). More than 60% of the respondents practiced in an urban setting with the remaining in more rural settings. The gender was overwhelmingly female (90.9%). Almost half (46%) had been in practice 10 year or less, another 40% in practice 11-20 years. Two-thirds of the participants were over age 40, with 39% over age 50. Overall, 82% of the respondents indicated that they utilized cancer screening guidelines. Of those, 91.3% used screening guidelines for breast cancer, 88.3% for cervical cancer, 90.3% for colorectal cancer, and 26.2% for other cancers. The most common source of guidelines was the American Cancer Society (73%) and the United States Preventive Service Task Force (68%). Other sources included the National Comprehensive Cancer Network and MD Anderson Cancer Center. Of the respondents who used cancer screening guidelines, more than 50% indicated that one of more of the guidelines was not appropriate for their practice. The most common reasons for not using guidelines included uncertainty about which guideline to use (10.3%), too many guidelines (10.4%), and changing guidelines that are difficult to keep up with (11.9%). A majority of respondents indicated that guidelines embedded in the electronic medical record (72.6%), an interactive tool (53.2%), and education about what guidelines are available (51.3%) would be helpful for utilization of guidelines. *Conclusion/Implications for Practice:* Although a low response rate may impact findings, overall results indicate that respondents are uncertain about which set of cancer screening guidelines to use and find it difficult to keep up with the updates. The respondents would like one set of simple and accessible guidelines that could be easily and automatically updated. A challenge will be to make the guidelines simple and easy to use, widely publicize their availability, and make them accessible and easy to update. 

## JL309. Development and Implementation of a Peer Review Process for APRNs

Marianne J. Davies, DNP, MSN, RN, APRN, CNS-BC, ACNP-BC, AOCNP, Yale University School of Nursing, West Haven, CT, and Katherine Tucker, APRN-BC, NE-BC, MSN, Yale New Haven Health, New Haven, CT

Peer Review is the evaluation of professionals by a peer who practices in a similar role and scope of practice. Yale New Haven Hospital (YNHH) Medical Staff Office utilizes ’The Joint Commission’ ongoing professional practice evaluation (OPPE) to conduct peer review for re-credentialing of all providers, following a traditional medical model. YNHH credentials more than 400 APRNs. The quality and depth of OPPEs varied by setting. The Chief Nursing Officer (CNO), the nursing representative on the Credentials Committee, recognized that APRNs were not involved in the process of review and APRN metrics were not incorporated into the evaluation as the historic tool had originally been developed for physicians. The CNO convened an APRN council to evaluate the current process and make recommendations for a more robust process. The APRN Council conducted a survey of credentialed APRNs to gain understanding of the needs of the group. The survey revealed that only 25 % of APRNs had reviews completed by another APRN. The remainder had evaluations done by physicians and administrators. APRNS expressed the need for increased participation in the review process, peer support, feedback and advocacy. APRNs indicated the need to integrate metrics that reflect the APRNs contribution to patient care and outcomes. The APRN council developed a Likert-like peer-review tool utilizing the 6 domains of competence outlined by The Joint Commission OPPE. The tool was to be completed by a peer, collaborating physician, and self. They would also be peer reviewed by the APRN and their manager. The documents were then to be submitted to the Medical Staff Office to be used in the re-credentialing process. The peer review process was piloted, over 6 months, in three divisions: Oncology, Heart and Vascular, and Certified Registered Nurse Anesthetist (CRNA) groups. APRNs, Medical Staff Office and CNO evaluated the process. Each felt the process provided a robust profile of the APRNs practice. Minor revisions of the tool were suggested including consolidating domain evaluation, chart review and goal setting onto a double side piece of paper. The new process was adapted and instituted for all divisions in the hospital. It will be incorporated into affiliated community practices. The development of a standardized tool and an integrated infrastructure assures that contributions of APRNs can be quantified and patient outcomes tracked. The new process provides an opportunity to promote self-regulation and improve upon the quality of care provided. Adoption of the process provides the ability to review common patient care themes, promote practice standards and stimulate APRN driven research initiatives. 

## JL310. Pilot Evaluations of Experiences of Early Stage Post-Surgical Lung Cancer Survivors

Kathleen Hopkins, PhD, RN, University of Pittsburgh, Pittsburgh, PA

*Background:* Over 220,000 patients are diagnosed with lung cancer each year. Although only 15% are diagnosed at an early stage, with the possibility of curative surgery, this population numbers over 165,000 annual survivors. The purpose of this study was to describe the first year lived experiences of these post-surgical early stage lung cancer survivors. *Research Approach:* Open-ended interviews. *Setting:*A large university based surgical cancer center located in the northeastern United States. *Participants:* 15 early-stage post-surgical lung cancer survivors. *Methodologic Approach:* Interpretive phenomenology based on Heideggarian hermeneutics and revealing the subjects’ post-operative lived experiences. *Main Research Variables:* The lived experiences and early stage lung cancer survivors within their first post-operative year, themes and patterns from these experiences. *Findings:* Although patients often did not complain, experience impacted patient and are described in 4 themes: (1) thankfulness of an incidental diagnosis, (2) surprise reactions to post-surgical procedures (chest-tubes, narcotics), (3) the annoyance of concurrent symptoms (pain, fatigue, mood disorders, cough, shortness of breath), and (4) acceptance and striving for a new sense of normalcy. *Conclusions:* A larger study to better investigate the experiences from diagnosis to survival of lung cancer survivors.

## JL311. Symptom Management Strategies for Patients Receiving Anaplastic Lymphoma Kinase (ALK) Inhibitors for Non–Small Cell Lung Cancer (NSCLC)

Jennifer E. Jacky, MSN, ARNP and Christina Baik, MD, MPH; Seattle Cancer Care Alliance, Seattle, WA

*Introduction:* Targeted therapies for the treatment of metastatic NSCLC are associated with a distinct set of adverse side effects that differ clinically from those seen with chemotherapies. Our institution has been actively involved in clinical trials of ALK inhibitors, and have gained extensive experience in managing patients receiving these agents. Symptom management (medication or non-medication based) is patient-centric and involves a multi-specialty team who share the goal of maintaining patients on appropriate palliative therapy. Here we present 3 cases illustrating symptom management strategies for ALK inhibitors, focusing on fatigue, anorexia, gastrointestinal toxicities, and transaminitis. *Discussion:* Case 1: A 79-year-old woman with ALK+ NSCLC was treated initially with pemetrexed followed by crizotinib. Crizotinib-associated nausea and diarrhea were managed with ondansetron, but activity-limiting fatigue was problematic. For fatigue we screened for insomnia, sleep apnea, depression, and polypharmacy. This patient benefited from low-dose trazodone with improvement of sleep and fatigue. She was treated with an antidepressant for depression/anxiety and referred to physical therapy for general strengthening, which enabled her to travel with family. At disease progression the patient initiated ceritinib and experienced an associated reduction in tumor burden in the chest and brain, resulting in improvement in chest pain, cough and dyspnea. Reduced appetite was problematic and thoroughly evaluated. The patient benefitted from referral to nutrition and increased ondansetron. Case 2: A 45-year-old woman with metastatic ALK+ NSCLC underwent spinal and whole-brain radiation. She initiated ceritinib 750 mg and dose reduced to 600 mg upon reporting significant diarrhea and prolonged QTc; nausea and emesis remained severe despite dose reduction. Good effect was found with oral ondansetron and atropine/diphenoxylate dosed 30 minutes before and prochlorperazine 30 minutes after taking ceritinib. This patient benefited from dosing at night, evaluation of psychosocial factors, management of reflux, close monitoring, and encouragement in taking medications. Case 3: A 60-year-old man presented with metastatic NSCLC, renal, and cardiac comorbidities. At confirmation of ALK+ disease he was started on crizotinib, followed by ceritinib upon progression. One month after starting ceritinib, tumor responses were seen in the chest and brain, but he developed grade 3 transaminitis. Repeated episodes of transaminitis have been managed with dose reduction, holding of drug until normalization of liver function, and biweekly monitoring of liver function with good ongoing tumor control. *Conclusion:* A comprehensive approach to managing symptoms associated with targeted therapies for metastatic NSCLC can enable patients to remain on palliative treatments for several years that are both tolerable and effective in controlling cancer growth. 

## JL312. Management of Lung Cancer Patients With Chronic Obstructive Pulmonary Disease (COPD) Receiving Nivolumab

Kim Hart, ANP, AOCN, Illinois Cancer Specialists, Arlington Heights, IL, and Marilyn Borkgren, APN, MS, CCNS, Suburban Lung Associates, Chicago, IL

*Introduction:* Earlier this year, nivolumab, a fully human IgG4 anti–programmed death-1 (PD-1) immune checkpoint inhibitor antibody, gained approval for advanced-stage squamous-cell lung cancer in patients whose disease progressed after treatment with platinum-based chemotherapy. Survival advantages demonstrated in clinical trials led to its early approval. Management of patients receiving immuno-oncology (I-O) agents such as PD-1 checkpoint inhibitors can be challenging. Many of these patients have COPD and other comorbid conditions, making it difficult to identify side effects specific to PD-1 pathway checkpoint inhibitors. *Description:* Initial and ongoing management of patients with COPD and lung cancer should include thorough evaluation and collaboration with a pulmonary team, early identification and severity staging of COPD, maximization of controller medications, up-to-date vaccinations, and prompt intervention for respiratory tract infections (eg, bronchitis, pneumonia, infectious pneumonitis). Global Initiative for Chronic Obstructive Lung Disease guidelines support pulmonary rehabilitation, smoking cessation programs, regular follow-up with a pulmonary team, and avoidance of long-term oral corticosteroids. While it is not known if steroids suppress antitumor activity, it is important to identify early and manage appropriately all immune mediated adverse events, including COPD exacerbation. Further exploratory studies are needed to assess how steroids used to treat immune-mediated adverse events or COPD exacerbations impact efficacy. Oncology and pulmonary nurse practitioners and physician assistants can provide important education to lung cancer patients with COPD regarding I-O therapies. It is essential for patients to understand the mechanism of action of I-O therapies, the differences in side effects compared with traditional chemotherapy, and the importance of managing comorbid conditions to maintain underlying health and therapy administration. These patients should undergo pulmonary evaluation before initiation of therapy. Consistent follow-up and collaboration with both oncology and pulmonary teams is the key to optimizing therapy for patients with lung cancer and underlying COPD, particularly given the importance of differentiating I-O–related pneumonitis from infectious pneumonitis in this population. Collaboration across both teams on the use of oral steroids for any reason is critical. *Summary:* Comprehensive management of comorbid pulmonary conditions such as COPD is necessary for patients to fully benefit from I-O therapy. While nivolumab is the first I-O therapy available to patients with advanced-stage squamous non–small cell lung cancer, other promising I-O therapies for lung cancer patients are in development, making this a hopeful time for patients with metastatic lung cancer. 

## JL313. By Nurses, For Nurses: A Successful Educational Intervention That Addressed the Clinical Challenges of Treating Elderly Patients With Multiple Myeloma

Patricia M. Repetto, MEd, MedscapeCME, New York, NY, Emily S. Van Laar, MS, Medscape, New York, NY, and Beth Faiman, PhD, APRN-BC, AOCN, Cleveland Clinic Taussig Cancer Institute, Cleveland, OH

*Introduction/Background:* Because multiple myeloma (MM) typically occurs in patients over 70 years of age, many patients present with comorbidities. This can present a challenge for the care team who must consider multiple factors which prevent these patients from being able to tolerate standard treatment protocols without dose modifications and supportive therapy. As a result, MM therapy for older patients requires a tailored approach to treatment selection and supportive care. *Materials and Methods:* A web-based educational program designed as a roundtable discussion among nurses and tailored to a nursing audience was posted on Medscape Oncology in September 2014 (http://www.medscape.org/viewarticle/831381). The effect of nursing-centric education was assessed using a pre-assessment/post-assessment study design. This study design compared participants’ responses to questions before exposure to educational content (pre-assessment) with the same participants’ responses to identical questions placed after the educational content (post-assessment measurement). A paired 2-tailed t-test was used to assess whether the mean pre-assessment score was different from the mean post-assessment score. A Pearson’s 𝝌₂ statistic was used to measure changes in responses to individual questions. Probability values (*P* values) were also calculated for both t-test and 𝝌₂ statistics to determine significance level (á). Individual learners were defined as "Improved" (higher post education score) "Knowledge Reinforced" (answered both pre and post education questions correctly) or "Unaffected"(pre and post scores answered incorrectly). Between September 25, 2014 and January 7, 2015, more than 22,000 nurses participated in the activity, of which 1415 were assessed. *Results:* Compared with the baseline assessment, an overall medium effect size of 0.724 was seen among nurses (*p* <.05). Specifically:

- 82% improved/reinforced understanding appropriate nursing practice for a new diagnosed patient with MM undergoing first line treatment using lenalidomide/dexamethasone regimen and developing a side effect (*p* <.05).
- 80% improved/reinforced knowledge on the benefit of the "air sandwich" technique as a strategy for reducing injection site reactions associated with subcutaneous administration of bortezomib (*p* <.05).
- 72% improved/reinforced knowledge related to proper carfilzomib dosing and relevant patient-caregiver communication (*p* <.05).
- 62% improved/reinforced knowledge related to the safety profiles of current treatments approved to treat patients with MM (*p* <.05).

*Conclusions:* This study demonstrated the success of a nursing-focused educational initiative designed by nurses to improve the care and management of elderly patients with MM. Remaining gaps include outlining best nursing practices that can minimize adverse events in MM patients and summarizing critical caregiving communication principles for MM patients.to lung cancer patients with COPD regarding I-O therapies. It is essential for patients to understand the mechanism of action of I-O therapies, the differences in side effects compared with traditional chemotherapy, and the importance of managing comorbid conditions to maintain underlying health and therapy administration. These patients should undergo pulmonary evaluation before initiation of therapy. Consistent follow-up and collaboration with both oncology and pulmonary teams is the key to optimizing therapy for patients with lung cancer and underlying COPD, particularly given the importance of differentiating I-O–related pneumonitis from infectious pneumonitis in this population. Collaboration across both teams on the use of oral steroids for any reason is critical. *Summary:* Comprehensive management of comorbid pulmonary conditions such as COPD is necessary for patients to fully benefit from I-O therapy. While nivolumab is the first I-O therapy available to patients with advanced-stage squamous non–small cell lung cancer, other promising I-O therapies for lung cancer patients are in development, making this a hopeful time for patients with metastatic lung cancer. 

## JL314. Patient Reported Symptoms, Concerns and Provider Intervention in Patients With Multiple Myeloma

Beth Faiman, PhD, RN, APRN-BC, AOCN, Cleveland Clinic Taussig Cancer Institute, Cleveland, OH, Paul Jacobsen, PhD, Moffitt Cancer Center, Tampa, FL, Gregory Garber, MSW, LCSW, Sidney Kimmel Cancer Center at Thomas Jefferson University, Philadelphia, PA, Alyssa M. Cadman, BSW, Philadelphia, PA, Stephanie Chapman, RN, Nadia Still, DNP, Berlin, NJ, SarahLena Panzer, BS, Carevive Systems, Philadelphia, PA, Karen Hammelef, DNP, RN, On Q Health, Dearborn, MI, Carrie Stricker, PhD, RN, Abramson Cancer Center at the University of Pennsylvania, Philadelphia, PA, and Rachid Baz, MD, Moffitt Cancer Center, Tampa, FL

*Introduction:* Multiple Myeloma (MM) treatment is undergoing rapid transformation. New agents and regimens create an increased need to proactively screen for, assess, and manage patient reported symptoms and concerns. The Carevive Care Planning System (CPS™) captures electronic patient-reported outcomes (ePRO) and clinical data to generate tailored, evidence-based symptom management and supportive care plans, promoting supported patient self-management. *Description:* 90 patients with MM will be enrolled at 3 sites in this pilot intervention study. Using a study-provided tablet in the waiting room prior to a clinic visit, MM patient engaged with the Carevive CPS to report symptoms and concerns resulting in an auto-generated care plan that is reviewed by the clinician, who approves or rejects recommendations at the point of care. The study’s primary aim is to examine provider use of evidence-based symptom care behaviors (education, recommended interventions) in study participants compared to historical controls. This analysis focuses on symptoms and concerns of intervention participants, as well as provider care behaviors for these individuals, determined by which evidenced-based recommendations were retained. *Summary:* To date, 38 MM patients on active treatment have enrolled. Patients ranged in age from 36-89 (mean age=60.4). 55.3% of patients (n=21) were on Dexamethasone, 42.1% (N=16) were on bortezomib (Velcade), and 39.5% (n=15) were on lenalidomide (Revlimid). Physical symptoms of fatigue, peripheral neuropathy (PN) and diarrhea were the most prevalent. 75% (n=27) of patients reported experiencing some level of tiredness/fatigue within the previous week, with 67% reporting a moderate to severe level (mean 4.7 (1-10 severity scale)). 60.5% (n=23) reported PN of whom, 69.6% (n=16) reported painful PN. 37% (n=14) reported diarrhea, of which 57% reported this symptom frequently-almost always. Patients reported being most concerned with: (1) making the right treatment decision (47.4%), (2) understanding (their) treatment plan (44.7%), and (3) managing fatigue (31.6%). 100% of care plan recommendations for patients experiencing moderate to severe levels of fatigue were accepted by providers and delivered to patients (i.e., consider starting a supervised exercise program), with 68% of the associated tasks being accepted and delivered (i.e., schedule an appointment with a physical/occupational therapist to develop an exercise plan). 92% of patients received a recommendation to learn about MM treatments and side effects with 100% acceptance and delivery. *Conclusions:* Physical symptoms were highly prevalent; largely of moderate to high severity. Providers instituted both evidence-based proactive and reactive symptom management strategies. Interestingly this included a high rate of acceptance for referrals to physical therapy to manage fatigue, highlighting the recognized role of cancer rehabilitation as a treatment strategy. Even with high symptom prevalence and severity, patient top concerns were predominantly understanding and making decisions about treatment; supporting the need to address treatment education and decision-making support with symptom management.

## JL315. The 2014 Mentorship Program: Bridging Communication and Educational Gaps in Multiple Myeloma

Beth Faiman, PhD, RN, APRN-BC, AOCN, Cleveland Clinic Taussig Cancer Institute, Cleveland, OH, and Sandra E. Kurtin, RN, MS, AOCN, ANP-C, The University of Arizona Cancer Center, Tucson, AZ

*Introduction:* Significant changes to the multiple myeloma (MM) landscape have occurred within the past 10 years. Keeping up-to-date on drugs to treat MM and the management of side effects is challenging. The 2014 Multiple Myeloma Mentorship Program (MMMP) was an online educational activity designed to bridge serial learning activities and independent clinical practice. The MMMP provided a focused, interactive, peer-to-peer educational experience for 10 mentees leading to Advanced Clinical Educator (ACE) status. The curriculum included activities focusing on survivorship, strategies for side effect management, and treatment of MM in first-line, maintenance, and relapsed/refractory settings. The Postgraduate Institute for Medicine and AXIS Medical Education, in partnership with RealCME, provided this mentorship opportunity. This activity was supported by an educational grant from Millennium: The Takeda Oncology Company. *Description:* From May 2014 to March 2015, 10 mentees, representing 9 states, were paired with 1 of 2 experienced MM mentors. The mentees were taught about MM with a structured serial learning curriculum, which included a baseline self-assessment, 2 self-study sessions, and 2 virtual summits. Each mentee then presented 2 MM-focused slide decks created by the mentors to 257 learners at various practice settings nationwide. Learners included nurses, physicians, and support staff. Each mentee then completed a final self-assessment to conclude their participation in the MMMP. The modular activities were also opened to a national audience of healthcare professionals (national curriculum). The objective of the program was twofold: to improve mentees’ knowledge, competency (case-based), confidence, and level of performance in the treatment of patients with MM; and to build their skills as MM subject matter experts and speakers. *Methods:* Pre-test and post-test surveys of the 4 learning domains were compiled across all the activities in the MMMP. All questions in the pre-test and post-test sections were tagged in the RealMeasure platform by question type and for particular learning objectives and subject areas. A RealIndex composite score, based on a multidimensional situation-based question that addresses the learning objectives of the curriculum, was also calculated to measure performance. *Summary:* For the mentorship cohort, significant gains were measured across the curriculum in learning domains of knowledge (change = 6%; *P* = .01), confidence (change = 11%; *P* = .001), and practice strategy (change = 25%; P < .0005). The gains observed in competence (change = 13%; *P* = .141) and on the RealIndex (change = 9%; *P* = .139) did not meet statistical significance due to small sample size. Upon completion of the MMMP, mentees achieved ACE status. Additionally, findings show that the mentorship cohort’s average scores were higher compared to those attendees participating in the national curriculum presentations at both pre-test and post-test in knowledge, confidence, and performance, demonstrating the success and impact of participation in the MMMP. 

## JL316. The APSHO Practice Survey

Sandra E. Kurtin, RN, MS, AOCN, ANP-C, The University of Arizona Cancer Center, Tucson, AZ, Pamela Hallquist Viale, RN, MS, CS, ANP, University of California, San Francisco, CA, Heather M. Hylton, MS, PA-C, Memorial Sloan Kettering Cancer Center, New York, NY, Christopher J. Campen, PharmD, BCPS, BCOP, Arizona Cancer Center, University of Arizona, and Wendy H. Vogel, MSN, FNP, AOCNP, Wellmont Cancer Institute, Kingsport, TN

*Background:* The Advanced Practitioner Society for Hematology and Oncology (APSHO) conducted a survey of its members over a 6 week period in the summer of 2014 to describe practice patterns. *Significance of the Problem:* Cancer survivors are projected to exceed 19 million by 2024. The oncology workforce is projected to fall short of the expected demand, yet with increasing complexity in providing oncology care. Integration of advanced practitioners (AP) in oncology using collaborative practice models is proposed as an ideal solution to the challenge of providing care to the growing cancer population across multiple settings given an anticipated shortfall of practicing hematologists and oncologists. *Results:* One-hundred ninety two responses with representation from 37 of 50 states were completed. The majority of respondents (56%) reported more than 10 years of oncology experience. Of these, 22% reported more than 20 years of experience. Twenty-three percent of respondents reported less than 5 years of experience. Average hours worked by APs in oncology was more than 40 hours per week (63%), with a minority working part-time (13% working 30 hours or less; 25% working 30-40 hours per week). The high numbers of hours work reflect the complexity of oncology care and the high numbers of oncology patients requiring care. The most common practice setting for APs in this survey was outpatient oncology settings with no bone marrow transplant coverage (76%). Models of care included blended models (39%, n=68) and independent visits (31%, n= 53), and practice within an interdisciplinary team (18%, n=32). This is not surprising given the highly experienced workforce represented in this study. Sixty-eight percent of APs in this survey worked with 1–5 physicians. *Limitations:* It is uncertain if these data represent the AP in oncology workforce as a whole, or represent APs that are motivated to engage in APSHO as a new organization focused on the AP in oncology and collaborative practice. *Conclusions:* advanced practitioners in oncology represent a diverse group of health care providers poised to fill the anticipated oncology workforce shortfall. Using a collaborative practice model, hematologists and oncologists together with APs in oncology have the opportunity to develop programs that will effectively address the complex needs of the cancer patient and their caregivers across the continuum of care. Organized programs to address educational and training needs of the APs will be necessary. Continued collaborative efforts among professional organizations that represent cancer providers is imperative. 

## JL317. Educational and Knowledge Gaps Among Community Healthcare Providers (HCPs) Treating Patients With Lower-Risk Myelodysplastic Syndromes (MDS)

Sandra E. Kurtin, RN, MS, AOCN, ANP-C, The University of Arizona Cancer Center, Tucson, Arizona, Joan Latsko, DNP, CRNP, OCN, AOCNP, Wheeling Hospital, Wheeling, West Virginia, and Elizabeth Finley-Oliver, BSN, RN, OCN, H. Lee Moffitt Cancer Center and Research Institute, Tampa, Florida

*Background:* MDS represent a heterogeneous group of myeloid malignancies with variable prognosis. MDS are underdiagnosed, and many lower-risk patients are offered only supportive care (Khan AM. Am J Med. 2012;125[7 suppl]:S15-S7). This study aimed to describe current treatment management of lower-risk MDS in the community setting and identify educational gaps among oncology professionals, including hematologists/oncologists, advanced practitioners (APs), and registered nurses (RNs). *Methods:* In 2014, three facilitated interdisciplinary meetings about the management of lower-risk MDS, including discussion concerning treatment of patients with lenalidomide, were held in Boston, Chicago, and San Francisco. Attendees included 16 hematologists/oncologists, 14 nurse practitioners, 2 physician’s assistants, and 7 RNs, from 14 US states, all with clinical experience managing patients with lower-risk MDS. Results presented exclude RNs. *Results:* Compared with hematologists/oncologists, most APs had < 10 years’ experience managing patients with lower-risk MDS (20% vs 75%, respectively). The key points regarding lenalidomide treatment that APs most often discussed with their lower-risk del(5q) MDS patients were adverse events (AEs; 88%), time to response (88%), and duration of treatment (75%). Most APs (56%) noted that patients with concerns about lenalidomide treatment contacted a nurse as their primary point of contact; 29% noted that their practice has a dedicated triage nurse, with APs or physicians being consulted if necessary. 37% of APs and 53% of hematologists/oncologists felt there were MDS topics that they or their colleagues required more information about. APs wanted more information about expectation and management of cytopenias, when to initiate treatment, time to response, new treatments, and implications of cytogenetic abnormalities. Hematologists/oncologists were interested in more information on prognostic scoring systems, lenalidomide for treating high-risk MDS, novel therapeutics, genetic mutations, and optimal sequencing of therapeutic agents. 81% of APs and 73% of hematologists/oncologists felt patients with lower-risk MDS needed more education about their disease. Topics mentioned by APs included AEs (38%), time to response (31%), compliance (25%), and MDS as a cancer (13%). Topics mentioned by hematologists/oncologists included treatment options and outcomes (20%), MDS as a cancer (20%), treatment of fatigue (7%), transfusion management (7%), and differences between management of higher- and lower-risk MDS (7%). *Conclusions:* These interdisciplinary meetings discussed the management of lower-risk MDS and identified gaps in HCP knowledge. There were certain topics about which HCPs, and their patients, required or requested additional information. These knowledge gaps may inform future educational and training sessions to ensure optimal treatment and patient management in lower-risk MDS. 

## JL318. Collaborative Interventions in the Treatment of Chemotherapy-Induced Oral Mucositis With Low Level Laser Therapy (LLLT)–A Case Series

Cheryl E. Jones, CRNP, AOCN, Cynthia Castillo, ND, FABNO, Marie Winters, ND, FABNO, and Alexandra Louden, ND, Cancer Treatment Centers of America, Philadelphia, PA

*Introduction:* Oral mucositis refers to the damage of the mouth’s mucosa resulting from chemotherapy or radiation therapy. It is one of the most significantly debilitating, dose-limiting acute toxicities of cancer therapy that not only compromises cure rates, but increases the costs of supportive care. Oral mucositis occurs in approximately 40% of patients receiving standard therapy with drugs that affect DNA synthesis, such as 5-fluorouracil, capecitabine, gemcitabine, and pemetrexed. Incidence of oral mucositis ranges from 10–66% in patients receiving anthracyclines, taxanes, or platinum-based regimens. The collaborative, multidisciplinary efforts of advanced practitioners and Naturopathic Medicine providers afford us the opportunity to present three clinical case studies of adult cancer patients who each developed oral mucositis, and were treated with LLLT as part of their integrative cancer treatment regimen. *Description:* Two patients with carcinoma of unknown primary origin and one patient with lung adenocarcinoma were treated with three separate cancer treatment regimens: a fluorouracil-based regimen, a taxane-based regimen, and a tyrosine kinase inhibitor (TKI) regimen. Each of the patients developed oral mucositis in the week(s) subsequent to receiving conventional cancer treatment, which manifested as soreness or pain of the oral cavity or ulceration of the oral mucosa. Each of the patients were treated with LLLT either daily until mucositis had improved more than 50% compared to initial presentation or once every three weeks as preventative measure in the development of oral mucositis. Subjective pain, functional impairment and side effects were recorded at each visit. Patients reported improvement in severity of mucositis, delayed mucositis development and enhanced mucositis recovery time. No adverse reactions were reported secondary to use of LLLT. *Discussion/Conclusion:* Low level laser therapy is beneficial in both treating and preventing chemobiotherapy-induced oral mucositis in these three cases. This therapy is well tolerated and can significantly improve quality of life by reducing oral discomfort related to inflammation and ulceration from various cancer treatment regimens. While the prior clinical trials supporting use of LLLT in patients with cancer were completed in patients either undergoing hematopoietic stem cell transplantation or chemoradiation for head and neck cancer, this therapy could be considered as viable supportive care therapy for all cancer patients experiencing oral mucositis. This case series supports the need for prospective clinical trials to further elucidate the benefit of this treatment in a boarder patient population.

## JL319. Management of Skin Adverse Events (AEs) Associated With Nivolumab and Ipilimumab in Patients With Melanoma: A Nursing Perspective

Melissa Thebeau, ARNP, AOCNP, Moffitt Cancer Center, Tampa, FL, Krista Rubin, NP, Massachusetts General Hospital Cancer Center, Boston, MA, Annette Gaul, NP, Matthias Hofmann, PhD, Julia Grimm, NP, and Alyona Weinstein, NP

*Introduction:* The advent of immune checkpoint inhibitors has substantially improved the treatment of advanced melanoma in the past 5 years. Ipilimumab was the first drug to prolong overall survival, while nivolumab and pembrolizumab have shown greater efficacy and less toxicity than ipilimumab and have been incorporated into the standard of care. However, these agents are associated with a unique side effect profile that requires prompt recognition and management. Skin toxicities are the most common and often earliest occurring drug-related AE. While general management algorithms are available, we provide herein practical guidance based on clinical experience to help identify and treat skin AEs associated with nivolumab and ipilimumab. *Discussion:* In recent phase III trials, drug-related skin AEs occurred in 29%–42% of patients receiving nivolumab, 54% receiving ipilimumab, and 59% receiving the nivolumab and ipilimumab combination. The most common drug-related skin AEs are pruritus (16%–19% nivolumab; 35% ipilimumab; 33% combination), rash (9%–22% nivolumab; 21% ipilimumab; 28% combination), maculopapular rash (3%–5% nivolumab; 12% ipilimumab; 12% combination), and vitiligo (5%–11% nivolumab; 4% ipilimumab; 7% combination). In clinical experience, skin AEs may appear 2–3 weeks following the first dose, but are most common after 2–3 doses. Before onset of AEs, baseline dermatology referral, patient education on prompt reporting of skin AEs, and skin hygiene prophylaxis are recommended. Pruritus and/or rash, often mild to moderate (NCI-CTCAE grades 1–2), may appear on the arms, upper chest, back, and legs. Grade 1–2 rash that the patient does not find bothersome may not require treatment. Moisturizers, and if necessary, topical antihistamines or corticosteroids are recommended for symptomatic treatment, with oral diphenhydramine at night to relieve pruritus. Hydroxyzine or triamcinalone cream/ointment may also be prescribed if there is no relief with over-the-counter methods. For severe (grade 3) rash, initiate oral corticosteroids and hold ipilimumab/nivolumab until the rash improves. In cases of disabling (grade 4) rash, intravenous corticosteroids and permanent discontinuation of immunotherapy as well as biopsy or dermatologic consult is recommended. Systemic corticosteroids should be tapered appropriately upon improvement of symptoms. *Conclusion:* Advanced practitioners play a critical role in the management of skin AEs associated with nivolumab and ipilimumab. Through awareness of typical time to onset and clinical presentation as well as knowledge of management options and their appropriate application, nurses and nurse practitioners can help optimize treatment with the new class of immune checkpoint inhibitors in patients with advanced melanoma. 

## JL320. Creation of Metrics for Advanced Practice Providers in an Academic Outpatient Hematology Oncology Practice

Suzanne McGettigan, MSN, CRNP, ANP-BC, AOCN, Elizabeth Gilbert, MS, PA-C, Victoria Sherry, MSN, CRNP-BC, AOCNP, Colleen H. Erb, MSN, CRNP, ACNP-BC, AOCNP, and Genevieve Hollis, MSN, CRNP, ANP-BC, AOCN, University of Pennsylvania, Philadelphia, PA

*Introduction:* Much of oncology care is now delivered through a team approach; understanding the potential benefits of the physician/APP collaborative unit, in addition to the value of the APP individually, has never been more important. A recent study demonstrated that 54% of oncologists work collaboratively with advanced practice providers (APPs) such as physician assistants (PAs) and nurse practitioners (NP). At the Abramson Cancer Center (ACC) 83% of the MDs collaborate with an APP. With the widening gap between the demand for oncology services and available providers, it is estimated that these numbers will continue to increase. Despite this clear upward trend, there are not benchmark metrics specific to the oncology APP provider that can be utilized to represent the value of APPs as medical oncology professionals. At the ACC we were not using any metrics for our outpatient APPs. Each APP had only a yearly, multi page generic evaluation with subjective comments and scoring from their collaborating physicians and their direct supervisor. *Description:* A team of outpatient APPs formed a committee with the aim to search the literature for an applicable panel of APP driven metrics to use with our hem/onc division. The team included APPs from medical oncology, hematology-oncology, internal medicine, and radiation oncology. One of the barriers with developing these metrics was trying to assist our Information Technology (IT) staff to understand the nuances of APP practice patterns. Through this metrics group, we learned that our colleagues in radiation oncology had made progress in this area. After collaborating with radiation oncology’s IT specialist and our electronic medical record (EMR) IT specialists, our metrics group was able to better understand how to use and develop our own discrete elements within the EMR to represent our APP metrics. During and since our pilot, our IT support team has been integral in the implementation of revisions to our reports. A dashboard of evidence-based metrics was created for the APPs in the Hematology Oncology outpatient department at the ACC at the University of Pennsylvania. The metrics chosen represent four different aspects of APP practice. Each category contains 1-5 metrics. Electronic reports on the quality and patient volume metrics were created and generated for monthly, quarterly, and yearly review. The four categories are: patient satisfaction, quality metrics for independent and shared office visits, financial impact, and professional development. Patient satisfaction is gauged through Press Ganey reports for provider-specific encounters. Quality metrics include chart closure rate, allergy and medication reconciliation rate, documentation of smoking status and plan for smoking cessation, and documentation of pain and plan for management of pain. Financial impact includes total practice volume, APP independent visit volume, shared visit volume, total billing charges/RVUs. Professional development includes publications, presentations, participation in research or cancer center/hospital-based quality improvement committees, mentorship, continuing education credits, conference attendance, scholarships/grants/awards or pursuing an advanced degree. *Summary:*It is important to measure and show the quality of our care and our productivity within collaborative oncology practices. Creating evidence-based metrics in a diverse set of categories for the APPs within our cancer center has started to better illuminate the significance of our contributions. These metrics have now provided the tangible framework necessary for us to demonstrate and improve on the quality of the care we give and to help us grow as oncology professionals. 

## JL321. Use of Flow Cytometry in Clinical Practice

Dawn M. Betters, PhD, RN, Frances Payne Bolton School of Nursing, Case Western Reserve University, Cleveland, OH

Advanced practice nurses have played a vital role in progressing and enhancing clinical practice. They have collaborated with many disciplines, including, but not limited to, medicine, biology, sociology, and psychology. These collaborations have allowed them to broaden their skills and fields of study. Advanced practice nurses have begun incorporating basic science methods into their clinical theory for the benefit of health promotion and education. Flow cytometry is a well established and powerful method used for effectively measuring attributes of cells in disease and other health conditions. Examples of flow cytometry uses include cancer diagnoses, identification of biomarkers for disease, and immune function of caregivers. Flow cytometry has proven to be an important tool in both the clinical and research setting. For over sixty years, this method has aided clinicians and researchers in the ability to diagnose, treat, and monitor disease activity. The importance of understanding flow cytometry and its methodology is valuable to advanced practice nursing. A broad overview of flow cytometry and its experimental design will be reviewed. This overview will include the fluidics that contain the cellular suspension of interest, the laser that detects the fluorochrome-labeled cellular suspension, the electronics that convert the photons into interpretable data, and lastly, the computer interface which generates the data. As advanced practice nurses continue to progress and develop more specific and valuable roles as healthcare providers, it is important for them to understand scientific methods used in clinical settings for patient reporting and testing. This understanding will allow advanced practice nurses to anticipate the treatment, understand the implications of the results, and prepare and educate the patient. 

## JL322. Overview of Adverse Events (AEs) Seen With Combination of Nivolumab and Ipilimumab

Alyona Weinstein, RN, MSN, FNP-BC, Memorial Sloan-Kettering Cancer Center, New York, NY, Matthew Burke, MBA, RN, MSN, APRN-BC, Yale University School of Medicine, New Haven, CT, Smita Ranjan, MSN, APRN, University of Louisville, Louisville, KY, Jackie Hodgetts, MSc, RN, NMP Christie Hospital, Manchester, UK, Mary Kate Kasler, MSN, ACNP-BC, DNP, Memorial Sloan-Kettering Cancer Center, New York, NY, RuthAnn Gordon, MSN, FNP-BC, OCN, Memorial Sloan-Kettering Cancer Center, New York, NY, Vanessa Reed, MS, AGPCNP-BC, OCN, Memorial Sloan-Kettering Cancer Center, New York, NY, Yelena Shames, MSA, ACNP-BC, CNRN, Memorial Sloan-Kettering Cancer Center, New York, NY, Nana Prempeh-Keteku, MS, ANP-BC, Memorial Sloan-Kettering Cancer Center, New York, NY and Karla Lingard, RNBSc (Hons), Royal Marsden Hospital, London, UK

*Introduction:* Ipilimumab and nivolumab are immune checkpoint inhibitors approved for advanced melanoma. According to clinical trials, combination of these agents has greater efficacy compared with monotherapy. However, both nivolumab and ipilimumab are associated with AEs likely related to general immunologic enhancement. Incidence of AEs, including severe AEs and those leading to discontinuation, is higher with the combination regimen than monotherapy. To ensure that patients receive optimal benefit from combination therapy, prompt assessment and treatment of AEs is essential. Purpose: We will present case studies describing management of toxicities in patients receiving nivolumab and ipilimumab combination therapy. *Discussion:* In case 1, a patient with conjunctival melanoma metastatic to lungs and cervical lymph nodes received nivolumab and ipilimumab combination in an expanded access trial. Grade 2 maculopapular rash following dose 1 was managed by holding the patient’s second dose, referral to dermatology for biopsy, and treatment with clobetasol 0.05% topical cream. Grade 2 creatinine elevation, occurring after dose 3 and following initiation of angiotensin receptor blocker (ARB) for pre-existing hypertension, was managed by increasing oral hydration followed by oral prednisone 60 mg daily, discontinuation of ARB, and referral to renal services. Creatinine normalized and the patient completed dose 4 of combination therapy. Repeat imaging revealed substantially decreased pulmonary metastases, decreased lymph node size, and no new adenopathy. In case 2, a patient with BRAF mutation-positive melanoma with lung, spleen, abdominal lymph node, and bone metastases received 4 doses of combination nivolumab and ipilimumab over 12 weeks followed by nivolumab monotherapy every 2 weeks in a phase I study. Grade 2 hypothyroidism (onset day 15 of nivolumab monotherapy) was managed with levothyroxine while continuing nivolumab. Later, grade 1 pneumonitis was managed by holding nivolumab, thoracic consultation, and biopsy. Hemolytic anemia (hemoglobin 8.7 g/dL) was managed with hospitalization, intramuscular vitamin B12, and later with a course of prednisone. Following increase in splenic lesions and renewed symptoms, the patient underwent surgical resection, splenectomy, and discontinuation of nivolumab. Response was -94% prior to surgical resection with no evidence of disease post-surgery. *Conclusion:* When administering immunotherapy, it is imperative to collect detailed medical history to establish baseline, inform monitoring, and determine etiology of symptoms. Furthermore, collaboration among a multidisciplinary team is essential to initiate appropriate therapy and optimize safety management. Advanced practice nurses are uniquely positioned to educate patients in early recognition of AEs and play an important role in establishing appropriate monitoring and open dialogue among services.

## JL323. CREAM Principles: The Advanced Nursing Role in the Management of Dermatologic Adverse Events to Anticancer Therapy

Kathryn Ciccolini, RN, BSN, OCN, DNC, Memorial Sloan Kettering Cancer Center, New York, NY

*Background:* Dermatologic adverse events (dAE) can occur during and after all anticancer treatment regimens such as, but not limited to, rashes, nail abnormalities hand and feet symptoms, alopecia, xerosis, pruritus, radiation dermatitis, new onset proliferative skin lesions, hypersensitivity reactions, mucositis, secondary skin infections, and graft versus host disease. Therapies include chemotherapy, targeted therapy, immunotherapy, radiotherapy, hormone therapy, therapeutic transplants, and surgery. Consequences of these dAE have various negative impacts on quality of life (QoL), psychosocial and physical impact, instrumental and self-care activities of daily living and financial health. Most importantly, dAE can lead to alteration or discontinuation of anticancer therapy and trials. The advanced nursing role in the management of these untoward dAE is integral in ensuring adherence to anticancer and dermatologic treatment and optimizing disease outcomes. *Methods:* PubMed was searched filtering to humans only and English language from 2005 to June 30th, 2015 resulting in 788 articles. Further, a trail of citations from similar and cited references were followed to ensure the search was all encompassing of the topic. Articles selected for review were dedicated to oncologic literature mentioning dermatologic adverse events resulting from anticancer therapy or cancer diagnosis itself. The selected articles also mentioned the role of nursing in the management of these conditions under the CREAM principle subheadings. *Findings:* Trending themes were extracted from 133 articles to create the CREAM principles: Communication, Referral, Education/Encouragement, Assessment and Management/Monitoring. Further, the unique clinical experience from the role of the oncodermatology nurse at Memorial Sloan Kettering Cancer Center has been interlaced in the principles. This is the first definition in the philosophy of the advanced oncodermatologic nurse caring for the patient experiencing all types of dermatologic adverse events to any anticancer therapy. *Discussion & Implications:* Advanced oncology nurses should be well versed in the encompassing role by developing highly specialized skills in the management of dermatologic adverse events to anticancer therapy during treatment and survivorship setting for all types of cancers. Future studies are essential to elucidate the indispensable role of the advanced nursing in the management of dAE to anticancer therapy and the impact on patient outcomes. 

## JL324. Identification and Application of Clinical Resources for Advanced Practitioners Supporting the Management of Neutropenia Associated With Palbociclib

Joanne C. Ryan, PhD, RN, Pfizer, New York, NY, Lynn Nicole Pfeuffer, MSN, CRNP, Allegheny Cancer Center, Pittsburgh, PA, Annamaria Crisan, Bpharm, MSc, Pfizer, New York, NY, and Kristi Kay Orbaugh, RN, MSN, RNP, AOCN, Community Reg Cancer Care Group, Indianapolis, IN

Integration of a first-in-class drug into clinical practice generates a need for information to support its safe and effective use. In the early days post-FDA approval, the amount of available information can be limited and knowing how to get information is critical. Several paths exist, including queries to the pharmaceutical company and the pertinent literature. Good clinical assessment/judgment based on the medical history and clinical status of a patient further dictates the appropriate actions to be taken. Palbociclib, a novel CDK 4/6 inhibitor, was approved in February 2015 in combination with letrozole for the treatment of postmenopausal women with estrogen receptor (ER)-positive, human epidermal growth factor receptor 2 (HER2)-negative advanced breast cancer as initial endocrine-based therapy for their metastatic disease. The most commonly reported adverse reaction is neutropenia. Herein we highlight the clinical information needs of advanced practitioners (APs) and resources for managing neutropenia in metastatic breast cancer patients on palbociclib using a hypothetical case study to illustrate. In the first six months on the market, Pfizer US Medical Information (USMI) received 1943 queries related to palbociclib, 540 (28%) of which were generated by nurses and pharmacists. Of these, the most frequent requests sought information related to the use of palbociclib in alternative disease or clinical settings, product availability, and safety. Neutropenia was the primary focus of these safety queries. USMI responded to practitioners with letters containing a concise summary of relevant information from the FDA label, publications, and applicable Pfizer data. Addressing why neutropenia may be related to the mechanism of action of palbociclib and how this toxicity may be managed through dose modifications are among the important practical information included in these letters. Additionally, opportunities exist to obtain a broader review of the clinical trial data and treatment recommendations by accessing key publications available in the public domain. Furthermore, clinical assessment (including a thorough medical/treatment history) and clinical judgment are central to the safe management of patients on any new therapy. Adherence to recommended monitoring guidelines and comprehensive patient education around signs and symptoms of infection can aid in the early identification of neutropenia, its management, and interventions for potential complications. The emergence of new and innovative therapeutics, such as palbociclib, requires APs to rapidly integrate evidence from multiple sources. Clinical trial data, management guidelines/recommendations, and clinical assessment findings provide clinicians with the critical information and practical details necessary to safely and effectively manage their patients.

## JL325. Pilot Trial of Homebound Stem Cell Transplantation at Memorial Sloan Kettering Cancer Center

Jill M. Vanak, PhD, ACNP-BC, AOCNP, BMTCN, Memorial Sloan Kettering Cancer Center, New York, NY, Christina Bello, Memorial Sloan Kettering Cancer Center, New York, NY, Payal Dixit, Victoria Nguyen, Heather Landau, MD, Memorial Sloan Kettering Cancer Center, New York, NY, and Sergio Giralt, MD, Memorial Sloan Kettering Cancer Center, New York, NY

System and patient-based barriers lead to the inability of individuals to access appropriate treatment and oncologic care. Establishment of a homebound hematopoietic stem cell transplantation (HSCT) program promotes increased access to care and decreased patient disparity. This research initiative seeks to expand care of a patient treated with HSCT into the home setting. The primary objective of the protocol is to assess the feasibility of performing all post-HSCT care for select patients with a diagnosis of multiple myeloma in New York City in the home environment. The program is considered feasible if no more than 10/15 patients are readmitted to the hospital within 21 days of transplantation. Secondary objectives include assessment of adverse events, patient and caregiver satisfaction, and accuracy of telemedicine.We report on the creation and establishment of a homebound HSCT program within a single academic medical center with an emphasis on the role of the advanced practice provider (APP) and registered nurse. This homebound program is the second within the US, and the first within an urban landscape that employs services by providers who are not a part of a home health licensed agency or institution. Recognition that analysis of early experiences can inform subsequent efforts by other institutions in developing like programs merits a comprehensive process review of the homebound initiative, specifically the role of the APP and RN within the context of the provision of care within a home environment. Identification of infrastructure and research needs and the outline of an implementation framework with standard operating guidelines for providers will assist in the introduction of similar programs within other institutions. Barriers to program establishment included limited evidence as to both the clinical and financial effectiveness of a homebound program, reimbursement issues with third-party payers, increased burden to providers within an institution not licensed as a home health agency, and legal counsel due to individual state regulations surrounding home care. Review of the training offered to providers will allow for insight and eventual establishment of best practices related to the training of providers within a homebound stem cell transplantation program. As the majority of program establishment and implementation is completed in isolation, the need for an openly accessible knowledge base and structured collaboration surrounding sharing of best practices regarding the provision of oncologic care in the home environment is evident. 

## JL326. Progress Towards Value in Healthcare: Implementation of Time-Driven Activity-Based Costing (TDABC) in Hematopoietic Stem Cell Transplantation

Jill M. Vanak, PhD, ACNP-BC, AOCNP, BMTCN, Memorial Sloan Kettering Cancer Center, New York, NY, Elaine Duck, RN, MS, MA, Memorial Sloan Kettering Cancer Center, New York, NY, Christopher Cheavers, Memorial Sloan Kettering Cancer Center, New York, NY, Erika Duggan, MS, Memorial Sloan Kettering Cancer Center, New York, NY, Mark Radzyner, MBA, JD, Paul Nelson, MBA, New York, NY, and Miguel-Angel Perales, MD, Memorial Sloan Kettering Cancer Center, New York, NY

*Background and Specific Aims:* As healthcare institutions transition to value-based payment systems, there is an increasing need to identify the true costs of delivering health services. The costing methodology Time-Driven Activity-Based Costing (TDABC) was established in 2004 with the goal of quantifying the true cost and profitability of a service line or procedure, providing accurate data to support clinical and administrative strategic decisions. The primary objective of the current project was two-fold: to assess the feasibility of implementing TDABC and to determine the accuracy of the costing information provided by TDABC for autologous transplantation (ASCT) for patients with a diagnosis of multiple myeloma. We report results of a pilot study using TDABC to analyze the bone marrow aspirate and biopsy procedure (BMABx), a procedure completed for all patients prior to and during the ASCT process. *Methods:* The TDABC process involves five distinct steps, including identifying resources used for the specific procedure being reviewed, defining costs of each resource, estimating the practice capacity of the resources, calculating the cost of personnel per time unit, and determining the time units required for the procedure, which result in the calculation of cost per procedure. *Results:* Process maps were developed for ASCT and the BMABx. A comparative analysis of three distinct models of care delivery was done: 1. Physician (MD) model: entire procedure is completed by an attending physician (current clinical practice); 2. Advanced Practice Provider (APP) model: clinic staffed by APP who completes procedure; and 3. Joint model: MD and APP are present. Reimbursement according to payer mix was included in the analysis. Although the APP model led to reimbursement payment loss per procedure performed, it proved to be the most cost effective model of care. Furthermore, based on 2000 procedures/year, we estimated that switching to an APP clinic would free 500 hours of MD time/year (conservative estimate of approximately 50% of calculated time). Presentation of this research resulted in clinical practice change, with implementation of an APP model within the Adult Bone Marrow Transplant Service. *Conclusions:* TDABC can be implemented to evaluate clinical processes, and can result in practice change based on accurate cost data. This methodology and findings from this pilot study are being extended to study of the comprehensive ASCT episode of care. 

## JL327. Survivorship Care Plans: Strategies to Enhance Patient Utility and Value

Carrie T. Stricker, PhD, University of Pennsylvania, Philadelphia, PA, Amanda Seltzer, MSW, University of Connecticut, Storrs, CT, Karen Hammelef, DNP, RN, On Q Health, Dearborn, Michigan, SarahLena Panzer, BS, Carevive Systems, Philadelphia, Pennsylvania, and Ellen Dornelas, PhD, Hartford Hospital, Hartford, CT

*Introduction:* A decade ago the Institute of Medicine recommended that every survivor receive a survivor care plan (SCP) yet despite endorsements of clinical merit, evidence is still evolving regarding SCP utility and value to patients. *Method:* This pilot enrolled 65 women (Mean (M) age 60 years, range 43-82) who recently completed active treatment for breast cancer (stage 0-III, M=5 months since diagnosis). Patients completed an electronic patient reported outcomes (ePRO) survey via the Carevive Care Planning System (CPS) which is combined with clinical data to electronically generate tailored care plans with survivorship and symptom specific recommendations (i.e. follow-up care, self-management, supportive care referrals). Nurse practitioners conducted a consultative survivorship visit during which patients received their care plan. Patients completed follow-up assessments approximately 6 weeks later. *Data Analysis/Results:* Study outcomes include patient-reported use and helpfulness of, and satisfaction (1-5 Likert scale) with, their SCP. N=41 follow-up surveys (63%) were completed to date. Patients reported high overall satisfaction (M=4.19) and 95% (n=37) would, "recommend other women receive a similar care plan after cancer treatment." Patients also reported using the care plans M=6 ways, the most prominent being; 1) reading/planning to read carefully (100%), 2) using/planning to use to help inform about symptoms (89%), 3) using/planning to use to find information online and to help in talking to health care professionals about concerns (84% each). There was a trend toward patients with a higher number of symptom recommendations (recommendations linked to ePRO) reporting higher usefulness of the SCP (number of ways used; *p* = .055; t = -.379). No correlation was found between usefulness and number of surveillance/risk recommendations (recommendations not directly linked to ePRO). SCPs were most helpful to patients by, 1) helping to make decisions about what tests to receive and when (76% of patients), 2) helping them talk to family about their cancer experience (74% of patients), and 3) helping them to make changes in diet and exercise (73% and 70%, respectively). *Conclusions:* Satisfaction with the SCP was high and patients reported a variety of ways in which their SCP was used and was helpful. By linking point of care symptom screening with survivorship care planning, there is potential to provide patients the most valuable SCP possible by combining broader survivorship concerns with active symptom needs. Further studies are needed to determine whether these tailored SCP actually improve patient outcomes. 

